# The position of mefloquine as a 21^st ^century malaria chemoprophylaxis

**DOI:** 10.1186/1475-2875-9-357

**Published:** 2010-12-09

**Authors:** Patricia Schlagenhauf, Miriam Adamcova, Loredana Regep, Martin T Schaerer, Hans-Georg Rhein

**Affiliations:** 1 University of Zürich Centre for Travel Medicine, Hirschengraben 84, University of Zürich, Switzerland; 2 F.Hoffmann-La Roche, Basel, Switzerland

## Abstract

**Background:**

Malaria chemoprophylaxis prevents the occurrence of the symptoms of malaria. Travellers to high-risk *Plasmodium falciparum *endemic areas need an effective chemoprophylaxis.

**Methods:**

A literature search to update the status of mefloquine as a malaria chemoprophylaxis.

**Results:**

Except for clearly defined regions with multi-drug resistance, mefloquine is effective against the blood stages of all human malaria species, including the recently recognized fifth species, *Plasmodium knowlesi*. New data were found in the literature on the tolerability of mefloquine and the use of this medication by groups at high risk of malaria.

**Discussion:**

Use of mefloquine for pregnant women in the second and third trimester is sanctioned by the WHO and some authorities (CDC) allow the use of mefloquine even in the first trimester. Inadvertent pregnancy while using mefloquine is not considered grounds for pregnancy termination. Mefloquine chemoprophylaxis is allowed during breast-feeding. Studies show that mefloquine is a good option for other high-risk groups, such as long-term travellers, VFR travellers and families with small children. Despite a negative media perception, large pharmaco-epidemiological studies have shown that serious adverse events are rare. A recent US evaluation of serious events (hospitalization data) found no association between mefloquine prescriptions and serious adverse events across a wide range of outcomes including mental disorders and diseases of the nervous system. As part of an in-depth analysis of mefloquine tolerability, a potential trend for increased propensity for neuropsychiatric adverse events in women was identified in a number of published clinical studies. This trend is corroborated by several cohort studies that identified female sex and low body weight as risk factors.

**Conclusion:**

The choice of anti-malarial drug should be an evidence-based decision that considers the profile of the individual traveller and the risk of malaria. Mefloquine is an important, first-line anti-malarial drug but it is crucial for prescribers to screen medical histories and inform mefloquine users of potential adverse events. Careful prescribing and observance of contraindications are essential. For some indications, there is currently no replacement for mefloquine available or in the pipeline.

## Background - the need for chemoprophylaxis

Malaria is often imported into industrialized areas classified "malaria free" due to migration and tourist travel to malaria endemic areas. Approximately 80-90 million travellers will visit malaria endemic areas annually. In particular, travel to Africa has increased by 10% and sub-Saharan Africa has seen a recent 13% growth in international tourist arrivals [1]. Some 30,000 travellers from industrialized countries are reported to contract malaria each year and between 1-4% of travellers who acquire *Plasmodium falciparum *malaria will die [2]. The trend in imported malaria cases documented in North America and Europe [3], shows an increasing proportion caused by the life-threatening *P. falciparum*. Moreover, the incidence of malaria in travellers is likely to be an under-estimate as it does not include those diagnosed and treated abroad and because it is estimated that 40-70% of imported malaria cases are not reported to health authorities [2].

Travellers to sub-Saharan Africa are most at risk of contracting malaria. Recent estimates suggest an attack rate of 302 in 100,000 travellers to West Africa compared to lower rates in Southern Africa 49/100,000 and much lower rates in Eastern Asia 5.4/100,000 and the Americas 1/100,000 [4].

Travellers who return to their country of origin to visit friends and relatives (VFR) have been shown to have a higher risk of acquiring malaria than regular tourists [5]. This is particularly true of migrant VFR travellers to West Africa [6].

The overall case fatality rate of imported *P. falciparum *malaria varies from 0.6 to 3.8% [2] but may be 20% or greater in the elderly or in cases of severe malaria even when optimally managed in modern intensive care units. Case fatality rates for malaria complicated by adult respiratory distress syndrome (ARDS) often exceed 80% [7]. However, malaria infection and associated fatalities are largely preventable. In nearly all reported fatal cases of imported malaria, travellers failed to use or comply with appropriate chemoprophylactic regimens. Recent reports of fatal cases of malaria in North America and Europe [8] highlight problems in these areas. In nearly all fatal outcomes, patients were using either no chemoprophylaxis or an inappropriate regimen, had a delay or errors in the diagnosis of malaria by physicians and laboratories, or received incorrect initial chemotherapy.

## Definitions

Malaria chemoprophylaxis can be defined as the use of anti-malarial medication to prevent the occurrence of the symptoms of malaria. No available drug can destroy the sporozoites (inoculated by the *Anopheles *mosquito), which remain only briefly in the bloodstream before entering the liver. Drugs which act on the parasite in the liver tissue are termed "*causal prophylactics*", for example, atovaquone and proguanil. Doxycycline has only a weak causal effect. "*Suppressive prophylactics*" or blood schizontocidal drugs act in the bloodstream when parasites invade the erythrocyctes. Most anti-malarial drugs fall into this category, for example, mefloquine and doxycycline.

### Current guidelines for malaria chemoprophylaxis

**Figure 1:**
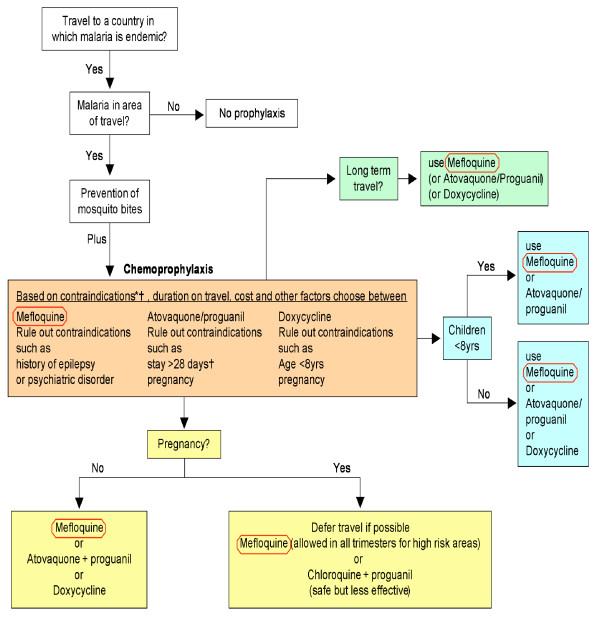
Malaria chemoprophylaxis for risk groups

There is a lack of international harmony in guidelines for malaria prevention (Table 1), but almost all authorities and expert groups agree that mefloquine, atovaquone/proguanil and doxycycline are "priority" anti-malarial medications for travellers (Figure 1) to areas of chloroquine-resistant *Plasmodium falciparum *(CRPF) [9-17]. Other possibilities for chemoprophylaxis include the use of chloroquine with proguanil (rarely recommended due to widespread chloroquine resistance) and primaquine, only occasionally recommended for the prophylaxis indication in Canada and the US [10,12] (Figure 1). The choice of medication depends on the risk of malaria at the destination, resistance, the profile of the traveller (contra-indications, underlying health conditions, purpose of travel such as VFR), the duration of travel and finally cost and adherence issues. The registration status of the anti-malarial medications is also a factor. In Japan [17] for example, mefloquine is the only registered anti-malarial drug (Table 1) and no anti-malarial chemoprophylaxis is registered for children aged less than 8 years.

**Table 1:** Medications licensed and recommended for malaria chemoprophylaxis by various countries

Medication approved and available for malaria chemoprophylaxis	United States	Canada	United Kingdom	France	Germany	Switzerland	Japan	Australia
Mefloquine	licensed	licensed	licensed	licensed	licensed	licensed	Licensed but not approved for use in children	Licensed but not approved for use in children <14 years old

Doxycycline*	for age ≥8 years	for age ≥8 years	for age ≥12 years	for age >8 years	Off Label	for age ≥8 years	-	for age >8 years

Atovaquone-proguanil	licensed	licensed	licensed	licensed	licensed	licensed	-	licensed

Primaquine	Only licensed for radical cure but is recommended as primary prophylaxis in CDC guideline	licensed	- not recommended for primary prophylaxis	-	-	not registered for primary prophylaxis	not registered for primary prophylaxis	-

Proguanil in combination with chloroquine	-	-	licensed	licensed °	licensed	licensed	-	licensed

Adapted from Chen L, Wilson M, Schlagenhauf [49]

+ = licensed for malaria chemoprophylaxis and available in country.

- = not licensed for malaria chemoprophylaxis or not available.

* In some countries, doxycycline is licensed for specific indications but not malaria chemoprophylaxis. In Germany it is often used for this indication but not licensed for malaria prophylaxis "OFF LABEL" use.

° A fixed combination of chloroquine (100 mg base) and proguanil (200 mg) is available as Savarine^® ^in France

### The priority anti-malarials: indications, administration and clinical use

Mefloquine is available for malaria chemoprophylaxis since 1985 in Europe, since 1990 in the USA [18] and has been used by more than 35 million travellers for this indication. Mefloquine is effective in the prevention of CRPF malaria, except in clearly defined Thai border regions of multidrug resistance. Today, the drug is used clinically as a 50:50 racemic mixture of the erythro isomers and all clinical studies with the drug have used this mixture. The drug is an effective schizontocide and is active against the blood stages of all malaria species that infect humans including the recently recognized fifth species, *Plasmodium knowlesi *[19,20]. The once weekly regimen is considered conducive to good adherence. It is a priority anti-malarial for travellers to high risk malaria endemic areas and can be used for long-term travellers, pregnant women, breastfeeding women, small children weighing >5 kg and is a popular choice for families visiting friends and relatives because of its low cost and once weekly administration. The main drawback in chemoprophylaxis is the risk of adverse events particularly neuropsychiatric adverse events (see later section). The recommended adult dose for chemoprophylaxis is 250 mg base weekly as a single dose (US 228 mg base). Adults weighing <45 kg and children >5 kg require a weekly dose of 5 mg base/kg.

The fixed dose combination Malarone^® ^is 250 mg atovaquone combined with 100 mg proguanil hydrochloride (or an equivalent dose based on body weight in children) has an overall anti-malarial efficacy of circa 95% [21] and is well tolerated by travellers [22] with the lowest frequency of adverse events. A major advantage of this combination is its causal prophylactic activity directed against the early liver stages of *P. falciparum *allowing discontinuation of the regimen 1 week after leaving the malarious area. The combination is not effective against the latent stages of relapsing malaria, such as *Plasmodium vivax*. Disadvantages are the high cost of the regimen and the limited experience and applicability in risk groups such as pregnant women, small children (in Europe, the combination is licensed for children >11 kg, in the US for children >5 kg) and long-term travellers.

Doxycycline is third priority anti-malarial and an adult dosage of 100 mg daily is >95% effective in malaria chemoprophylaxis and has a weak activity against malaria liver stages. Doxycycline monohydrate is better tolerated than the older form doxycycline hyclate [23], which is associated with a 6% withdrawal rate due to gastrointestinal adverse events. Serious adverse events are rare [24]. It is similar in cost to mefloquine. Adherence to daily doxycycline is essential to ensure effectiveness and non-compliance is the main reason for prophylactic failures. Doxycycline is not recommended in pregnancy, in breast-feeding women and in children aged less than 8 years (in the UK in children aged less than 12 years) [9-11,13]. There are few data on the long-term use of 100 mg doxycycline malaria prophylaxis.

Differences exist among the chemoprophylaxis regimens that are licensed, recommended, and distributed in each country. Table 1 lists the key medications recommended for malaria chemoprophylaxis in the USA, Canada, UK, France, Germany, Switzerland, Japan, and Australia, and illustrates some differences among the guidelines. Even when the same medications are licensed and available, health authorities may differ in their assessment of risks, leading to different recommendations for the identical traveller and itinerary. In all guidelines worldwide, mefloquine is recognized as a key chemoprophylaxis option.

### Clinical use of mefloquine versus alternative chemoprophylactic agents

The ideal chemoprophylactic medication should be highly effective, cause few or no adverse events, be indicated for all travellers including pregnant women, nursing women, small children, long-term travellers, should be cheap and easy to use and should be registered globally for this indication. Currently no anti-malarial satisfies all the criteria for a "perfect" chemoprophylaxis regimen. Table 2 shows the position of mefloquine and other anti-malarial chemoprophylaxis regimens in terms of applicability to risk groups. Apart from real or perceived poor tolerability, mefloquine satisfies many criteria and for some groups such as pregnant women, there is no alternative. For many VFR families, who travel for long periods to high risk areas, cost can be a major factor in the decision as to whether or not to use chemoprophylaxis and if yes, which one. The low cost of weekly mefloquine plays an important role and recent research has shown that mefloquine chemoprophylaxis for travellers to West Africa (mainly VFR travellers) can be cost beneficial in terms of malaria cases avoided [25].

**Table 2:** Current malaria chemoprophylactic regimens based on their applicability for risk groups

Drug	Efficacy°	Tolerability	Long-term travel	Pregnancy	Breastfeeding	Small children <8 yrs	Cost*
Mefloquine	+++	+§	+++	+++	+++	+++	+++

Atovaquone/Proguanil	+++	+++	++	-	+++	+++	+

Doxycycline	+++	+++	++	-	-	-	+++

Chloroquine/Proguanil	+	+	+	++	++	+	++

° Efficacy of less than 75% gives a score of +, efficacy over 90% gives a score of +++ (Efficacy scores valid except for limited multi-drug resistant areas on the Thai-Cambodian and Thai-Myanmer borders)

*The lower the cost, the higher number of +, Weighting is arbitrary and can be modified to specific travellers and itineraries. In the table, emphasis is placed on risk groups.

§ Perceived tolerability is poor but serious adverse events are rare

### Efficacy of mefloquine prophylaxis in travellers

Mefloquine is recognized as an effective malaria chemoprophylaxis for travellers to high risk CRPF areas [18]. The drug is effective as a schizontocide, active against the blood stages of all malaria species that infect humans, including *Plasmodium knowlesi *[19,20]. The first report of mefloquine resistance came from Thailand in 1982 and this region remains a focus of resistance particularly on the Thai-Cambodian and Thai-Burmese borders where prophylaxis breakdown has been observed. As reviewed by Mockenhaupt, reports of mefloquine treatment or prophylactic failures have been reported from distinct foci in Asia and to a lesser extent, from Africa and the Amazon Basin in South America [26].

### Prophylactic failures and resistance

In many geographic regions, mapping of prophylactic failures, mainly in non-immune individuals has been used to detect early resistance development although it should be emphasized that prophylactic failures do not prove resistance. Mefloquine blood concentrations of 620 ng/ml are generally considered necessary to achieve 95% prophylactic efficacy [27]. As defined by Lobel, a prophylactic failure is defined as a confirmed *P. falciparum *infection in persons with mefloquine blood levels in excess of this protective level. Using this definition, an analysis of 44 confirmed *P. falciparum *cases, acquired in sub-Saharan Africa showed five volunteers with mefloquine-resistant *P. falciparum *malaria [27]. Other confirmed cases were attributed to poor compliance and the authors concluded that prevalence of mefloquine-resistant malaria in sub-Saharan Africa is still low. With regard to cross-resistance, there is recent evidence that exposure of parasite populations to anti-malarial drug pressure may select for resistance not only to the drug providing the pressure but also to other novel drugs. This was shown in Northern Cameroon, West Africa, where the detection of a high level of resistance to mefloquine was attributed to cross resistance with quinine [28], a drug that had been used in the area. Resistance to mefloquine appears to be distinct from chloroquine resistance, as shown by the activity of mefloquine against CRPF and by the inefficacy of verapamil to reverse mefloquine resistance. Moreover, *in vitro *studies have documented an inverse relationship between chloroquine and mefloquine resistance. Mefloquine resistance is associated with halofantrine resistance [29] and quinine resistance [30]. Penfluridol, a psychotropic drug has been reported to reverse mefloquine resistance in *P. falciparum in vitro *[31].

Innate resistance is still controversial and may to some extent be explained by cross resistance to other drugs [26]. The molecular basis of mefloquine resistance is currently unknown but may be the result of mutation or amplification of certain gene products, such as *Pgh1*, an energy-dependent transporter encoded by the *mdr *(multi-drug resistant) homolog *Pfmdr1*. Later studies demonstrate that mutations in *Pfmdr1 *may confer mefloquine resistance to sensitive parasites [30].

In summary mefloquine is recognized as a highly effective malaria chemoprophylaxis for non-immune travellers to high risk areas of chloroquine-resistant *Plasmodium falciparum *with the exception of clearly defined areas of multi-drug resistance mainly limited to Thai border areas.

### Use of mefloquine in groups at high risk of malaria

#### Pregnancy

Falciparum malaria in a pregnant woman poses significant risks for the mother, foetus and the neonate. A study of imported malaria cases from 2002 showed that 7% of imported malaria cases in the USA occurred in pregnant women of whom only 28% reported taking a recommended malaria chemoprophylaxis [31]. In France, the country with the highest number of imported malaria cases, 3-5% of all cases occur in pregnant women [32]. Women who have little or no immunity such as non-immune travellers are prone to episodes of severe malaria leading to stillbirths, spontaneous abortions or even maternal death. Foetal and peri-natal loss is estimated to be as high as 60-70% in non-immune women with malaria [33]. Travel by pregnant women or women who might become pregnant to destinations where chloroquine resistant *P. falciparum *(CRPF) malaria is transmitted should be avoided or deferred when possible.

Travellers to areas of chloroquine resistant *P. falciparum *need effective strategies to prevent malaria. Protection against mosquito bites is essential and a combination of protective measures including insecticide treated bed nets and DEET-(N, N-diethyl-3-methylbenzamide), containing repellents are considered safe in pregnancy and are recommended although the efficacy of these measures for pregnant women has not been unequivocally proven. Chloroquine and proguanil can be used by pregnant women but because chloroquine resistance is widespread, this combination has limited applicability. For high risk areas of chloroquine resistant *P. falciparum*, an effective chemoprophylaxis is imperative and the choice is a difficult one. Doxycycline is not recommended in pregnancy mainly based on the experience that tetracyclines cross the placenta and can lead to disturbances of skeletal growth, permanent discoloration of teeth, corneas and lenses [34]. Due to insufficient data, the combination atovaquone/proguanil (Malarone^®^) is not recommended in pregnancy although proguanil is widely used and considered safe in pregnancy and no teratogenicity has been observed in animal studies using atovaquone. Because of ethical and safety restrictions, few anti-malarials have been evaluated for pregnant, non-immune travellers [35] and a Cochrane review concluded that reliable research about the benefits and harms of treatments for malaria in pregnant women is scarce [36].

Currently, therefore, mefloquine is the only option for pregnant women who cannot defer travel and who need an effective chemoprophylaxis when visiting chloroquine resistant malaria endemic areas. Literature reviews and post-marketing surveillance studies of the Roche Drug Safety Database [32,37] have been positive for mefloquine use, even in the first trimester and have shown a low birth prevalence <4.5% of congenital malformations in women exposed to mefloquine (as Lariam^®^) during pregnancy. This level is within the background population level of congenital malformations estimated at between 5-6% [38].

These data have allowed expert groups to recommend mefloquine during pregnancy although a survey of the recommendations made by expert groups shows a variation in the advice given (Table 3). The WHO, UK and Swiss guidelines [9,11,13] sanction the use of mefloquine during pregnancy if there is travel to a high risk area but are more restrictive in the first trimester. The US and Canadian guidelines [10,12] now allow the use of mefloquine in all trimesters by pregnant women who cannot defer travel and who are at risk of contracting malaria. This decision was based on the view that the body of evidence showing that mefloquine is safe in pregnancy is adequate. Furthermore, the American and Canadian advice does not specify the need to avoid pregnancy for three months post exposure to mefloquine.

**Table 3:** Position of expert guidelines regarding the use of mefloquine in pregnancy

Expert Group	Recommendation
Manufacturer's label. International standard prescribing information	Mefloquine should be used during the first trimester only if the expected benefit justifies the potential risk to the fetus. Women of childbearing potential should be advised to practise contraception during malaria chemoprophylaxis with mefloquine and for up to 3 months thereafter. However in the case of unplanned pregnancy, malaria chemoprophylaxis with Lariam^® ^is not considered an indication for pregnancy termination.

UK Health Protection Agency Advisory Committee on Malaria Prevention for UK Travellers	Mefloquine should only be used in pregnancy if the need for it is great*. Women capable of childbearing should take contraceptive precautions while taking mefloquine and for three months after the last dose. Having taken mefloquine inadvertently during pregnancy is usually not viewed as an indication to terminate a pregnancy.

World Health Organisation	Mefloquine can be used in the second and third trimester. Pregnancy should be avoided for three months after use of the drug.

Centers Disease Control (CDC) (US expert group)	Use is permissible in all trimesters by pregnant women traveling to areas with known CQ-resistant *P. falciparum *when travel cannot be deferred. There is no clause suggesting that pregnancy should be avoided for three months following mefloquine use.

Committee to advise on tropical medicine and travel (CATMAT) (Canadian expert group)	Use is permissible in all trimesters by pregnant women traveling to areas with known CQ-resistant *P. falciparum *when travel cannot be deferred. There is no clause suggesting that pregnancy should be avoided for three months following mefloquine use.

SWISS working group	Mefloquine should be used during the first trimester only if the expected benefit justifies the potential risk to the foetus. Women of childbearing potential should be advised to practise contraception during malaria chemoprophylaxis with mefloquine and for up to 3 months thereafter.

(* An electronic update of the UK guidelines states the following: " *After expert advice, mefloquine may be considered for use in the first trimester of pregnancy***"**

http://www.hpa.org.uk/infections/topics_az/malaria/menu.htm)

#### Breastfed infants and small children

Young children are at special risk for malaria because of their inability to protect themselves from mosquitoes, the difficulty in administering anti-malarial drugs and the rapidity at which they become severely ill. Parents and guardians must pay particular attention to insect protection measures including repellents and treated bed nets.

Malaria chemoprophylaxis in the very young infant is a challenge. Although most anti-malarials taken by the mother will be present in breast milk, the drug concentrations are not considered high enough to provide an adequate protective dose for the infant.

With regard to breastfeeding, chloroquine, atovaquone/proguanil and mefloquine are considered compatible with breastfeeding. Proguanil is excreted into human milk in small quantities and in a rat study, atovaquone concentrations in milk were 30% of the concurrent atovaquone concentrations in the maternal plasma. Doxycycline is not routinely recommended.

The use of chloroquine prophylaxis is allowed in very small children but use is limited by widespread resistance to the drug. Atovaquone/proguanil prophylaxis (as paediatric tablets) can be used for children weighing more than 11 kg in Europe and in the US for children >5 kg according to new CDC guidelines [10]. Doxycycline is for children aged >8 yrs (in the UK doxycycline is only allowed for children aged >12 years). Mefloquine can be used for children >5 kg. This anti-malarial is well tolerated in children with good adherence due to simple once weekly dosage. The drug is bitter and parents should disguise the taste using chocolate or yoghurt. The tablets can be easily cut or broken and this regimen is very suitable for children who are travelling for long periods of time. Mefloquine is not approved for use in young children in Japan [17] or in Australia [16]. The findings of the studies done in children indicate a predictable pharmacokinetic profile of mefloquine in children, which is similar to that observed in adults [39]. The main age related difference in pharamacokinetics is that clearance per body weight is higher in older children aged 5-12 years compared to younger children aged 6 to 24 months [39,40].

### Long-term travellers

A review of the literature shows that 15-82% of long-term travellers, who spend lengthy periods in Africa, will report malaria [41]. In high-risk areas, the probability of being infected by malarial sporozoites increases to almost 100% when the stay in the endemic area exceeds four weeks with an average of 10 bites per night assuming that 1% of bites are infective [42]. This crude, statistical extrapolation underlines the necessity for exposure and prevention strategies during prolonged travel in malaria-endemic areas.

The terminology "long-term" usually refers to non-immune travellers who visit malaria endemic areas for a period of six months or longer. Typical long-term travellers include urban expatriates (such as diplomats, IT personnel), rural expatriates (mining workers, missionaries, humanitarian), military groups, Peace Corps volunteers, students, backpackers and occupational travellers [41]. Malaria prevention for these groups presents enormous challenges and there are few data on long-term use of anti-malarials. Studies on Peace Corps volunteers in West Africa provide the most robust data [27]. Some studies exist on long-term use of malaria prophylaxis and point to adherence with medication as a major stumbling block. Doxycycline, used for treatment of skin infections for months, can also be used as malaria prophylaxis with daily dosing, but side effects, especially vaginal candidiasis in women and photosensitivity is a problem in long-term users. Another option is the use of daily atovaquone/proguanil. In many countries outside of North America, use of the atovaquone/proguanil combination is limited to a period of 28 days. Data on the long-term use of doxycycline and atovaquone/proguanil are limited although some data are available on the use of atovaquone/proguanil chemoprophylaxis for periods longer than twenty weeks [43-45]. Long-term daily dosing is considered a risk factor for non-adherence. Mefloquine is a chemoprophylaxis regimen that has been adequately evaluated in the long-term setting and is a good option if well tolerated. There is documented long-term use of this medication in Peace Corps groups who took mefloquine for period of more than one year in West Africa [27] and were effectively protected against malaria in this high risk area, with a very low overall discontinuation rate due to adverse events (0.9%). They also noted that the frequency of reported adverse events decreased with prolonged use suggesting that mild symptoms are well tolerated. Pennie and colleagues reported on the steady state pharmacokinetics of weekly mefloquine in long-term travellers [46] and showed that toxic accumulation of the drug did not occur during prolonged weekly dosing. Mefloquine chemoprophylaxis use is well documented in long-term travellers and if well tolerated, can be used for prolonged periods [47]. The long half-life of the drug allows for weekly dosing and thus good adherence [48].

### Performance impact of mefloquine - use of mefloquine while flying, driving or diving

Because many reports highlighted neuropsychological mefloquine events, concern emerged that the use of mefloquine prophylaxis may impair performance and precision while driving, operating machinery or for soldiers in combat situations. For travellers, the impact of mefloquine on driving performance, in particular, required clarification. A review of mefloquine clinical toxicity studies including a study on driving performance with alcohol challenge showed that mefloquine, with or without small quantities of alcohol, does not impair driving [49]. Additionally, some diving schools prohibit the use of mefloquine although there is no scientific basis to support this ban. These controlled studies suggest that although mefloquine is associated with neuropsychological events in travellers, there is no performance deficit in persons who tolerate the drug [49].

### Malaria chemoprophylaxis for senior citizens

Older travellers are less likely to report adverse events with mefloquine than their younger counterparts [50]. One study specifically compared tolerability in senior travellers (>60 years) and younger travellers and found 9.7% of older travellers reported adverse events attributed to their anti-malarial medication compared to 13.6% in the (20-59 years) age group (p < 0.05) [50].

### VFR travellers

Travellers visiting friends and relatives (VFR), mainly immigrants and their children returning to their home country for vacations, are at particularly high risk for largely preventable infectious diseases such as malaria. In Western Europe, there are currently 20 million foreigners of which the majority are settled immigrants. One third come from a country outside of Europe. Increased global mobility and eased travel as well as the growing number of immigrants from malaria endemic countries have contributed to increased numbers of imported malaria cases in Europe. The immigrants from malaria endemic countries constitute a special risk group with high levels of malaria importation because they visit high risk areas for prolonged periods, are less likely to stay in air-conditioned hotels and often their status and financial circumstances hinder access to malaria chemoprophylaxis [25]. Mefloquine offers a reasonably priced option for financially constrained individuals or families who visit high-risk endemic areas. A recent analysis showed that mefloquine was cost-effective for travellers to high-risk areas to West Africa [25].

### Mefloquine and interactions

Mefloquine should not be used concomitantly with quinine or halofantrine and other structurally related anti-malarials or with medications that have central or peripheral nervous system activity. These interactions are well described in the labelling. A retrospective analysis of a database of anti-malarial tolerability data showed that other co-medications commonly used by travellers (such as anti-diarrhoea medication) have had no significant clinical impact on the safety of prophylaxis with mefloquine [51]. Mefloquine is extensively metabolized in the liver by CYP3A4, therefore, caution should be exercised when mefloquine is concomitantly administered particularly with potent CYP3A4 inhibitors such as ketoconazole. The co-administration of mefloquine with cardioactive drugs might contribute to the prolongation of QTc intervals, although, in the light of the information currently available, co-administration of mefloquine with such drugs is not contraindicated but should be monitored. Vaccination with oral live typhoid or cholera vaccines should be completed at least three days before the first dose of mefloquine.

Mefloquine and its metabolite are not appreciably removed by haemodialysis [52]. No special dosage adjustments are indicated for dialysis patients to achieve concentrations in plasma similar to those in healthy volunteers.

### Tolerability of mefloquine and adverse events

There is considerable controversy among international experts regarding the tolerability of mefloquine prophylaxis versus alternative regimens, such as doxycycline, chloroquine/proguanil and, the combination atovaquone/proguanil.

Adverse events can be divided into common events, usually mild and affecting large percentages of users and rare events, which are seen much less frequently and often recognized only after millions of users have used the drug. Rare events are usually not discovered in phase III trials and rely on post-marketing surveillance, which make the true incidence very uncertain as unreported events are likely.

There is only one double blind, randomized controlled trial [22] which compared all current malaria prophylactic regimens and analysed adverse events in 623 travellers randomized to atovaquone/proguanil (Malarone^®^), mefloquine (Lariam^®^), doxycycline (Vibramycin^®^) and chloroquine plus proguanil (Savarin^®^). Forty five percent of chloroquine and proguanil users reported mild to moderate adverse events, 42% of mefloquine users, 33% of doxycycline users and 32% of atovaquone/proguanil users. Significant adverse events (that interfered with daily activity) were reported in 11% of mefloquine users, 12% in chloroquine plus proguanil users, 6% in doxycycline users and 7% in atovaquone and proguanil users. Thus all malaria chemoprophylaxis regimens are associated with adverse events and many of the differences between the arms were not significant. It should be emphasized that an adverse event is not necessarily attributable to the anti-malarial drug, but reflects all intercurrent events experienced during the use of the drug.

An overview of the studies and databases comparing use of malaria chemoprophylactic agents in travellers (Tables 4 and 5) shows largely disparate results due to differing designs, definitions and methodologies and differing study populations. Regarding the reporting of any AE, the incidence during use of mefloquine is usually equivalent to the incidence reported for almost all chemoprophylactic regimens. However, women, in particular, were significantly more likely to experience adverse events [22,53,54].

**Table 4:** Adverse events interfering with daily activity (% of users)

Study	Population	Mefloquine	**Doxycycline **^°^	Atovaquone Proguanil	Chloroquine Proguanil
Phillips 1996	Australian	11.2	6.5	-	-

Schlagenhauf 1996	Swiss	11.2	-	-	-

Barrett 1996	UK	17	-	-	16

Steffen 1993	European	13	-	-	16

Hogh* 2000	International	-	-	0.2	2.0

Overbosch* 2001	International	5.0	-	1.0	-

Schlagenhauf 2003	International	10.5	5.9	6.7	12.4

Adapted from Schlagenhauf P [18]

* stopped taking anti-malarials

^° ^more AE with hyclate than monohydrate

**Table 5:** Incidence of SERIOUS# Adverse Events during Chemoprophylaxis

Report	Population	Mefloquine	Doxycycline	Atovaquone Proguanil	Chloroquine Proguanil
MacPhearson 1992	Canadian	1/20,000	?	?	-

Steffen 1993	European	1/11,000	?	?	1/5000

Croft 1996	UK soldiers	1/6,000	?	?	-

Barrett 1996	UK	1/600	?	?	1/1200

Roche Drug Safety 1997	Worldwide	1/20,000	?	?	-

Adapted from Schlagenhauf P [18]

# Hospitalization

### Withdrawal rates

Although often a subjective report by the traveller, when some measure of severity is applied to AE reporting, it appears that between 11-17% of travellers using mefloquine and other anti-malarials are, to some extent, incapacitated by adverse events. The extent of this incapacitation is often difficult to quantify and a good measure of the impact of adverse events is the extent of chemoprophylaxis curtailment or withdrawal from chemoprophylaxis. In a study of 5,120 Italian soldiers, deployed in Somalia and Mozambique in 1992-1994, the rate of prophylaxis discontinuation in chloroquine/proguanil users was 1.5% compared with a significantly lower rate of discontinuation in mefloquine users (0.9%) [55]. A controlled four-arm, double-blind, study showed intermediate withdrawal rates for mefloquine (3.9%) and doxycycline (3.9%) versus chloroquine/proguanil (5.2%) compared with atovaquone/proguanil which had the lowest withdrawal rate (1.8%) [22].

### The 2009 Cochrane Report

The latest Cochrane report [56] used eight controlled trials (n = 4240 participants) to evaluate current drugs (mefloquine, atovaquone/proguanil, doxycycline, chloroquine/proguanil) used for preventing malaria in travellers. No serious adverse events were reported in the users of any chemoprophylaxis. With regard to tolerability, the authors found that the chloroquine/proguanil regimen was the most poorly tolerated and had the most gastro-intestinal adverse events while atovaquone/proguanil and doxycycline had fewer neuropsychiatric adverse events than mefloquine. Overall the atovaquone/proguanil combination had fewer adverse events than mefloquine (RR 0.72), fewer gastrointestinal type adverse events (RR 0.54) and fewer neuropsychiatric adverse events (RR 0.86) and a better Total Mood Disturbance (TMD) score compared to mefloquine. The authors concluded however, that the quality of available evidence on tolerability is poor and that other factors such as cost, ease of administration, type of traveller and itinerary are also key factors in choosing an appropriate anti-malarial.

### Serious adverse events

These are adverse events that constitute an apparent threat to life, which require or prolong hospitalization or which result in severe disability. Steffen's large cohort study in travellers to East Africa estimated that one in 10,600 mefloquine users to have a serious adverse event (53). The study observed five probably mefloquine associated hospitalizations: two cases of seizures, 2 psychotic episodes and one case of vertigo. The rate of such events for chloroquine/proguanil users was 1/5,100 [57].

Data on serious adverse events only become available after widespread use of the anti-malarial and data is available for older drugs, such as mefloquine and chloroquine.

In one retrospective cohort analysis, serious neuropsychiatric AEs involving hospitalization were noted for 1:607 mefloquine users versus 1:1,181 chloroquine-proguanil users [58].

The British army's experience with mefloquine prophylaxis found the incidence of severe neuropsychiatric reactions to be ≤1:6,000 [59]. The most recent and comprehensive evaluation of serious events was a US analysis of hospitalizations [48] that found no association between mefloquine prescriptions and serious adverse events (as measured by hospitalizations) across a wide range of outcomes including mental disorders and diseases of the nervous system.

### Neuropsychiatric adverse events

This is the main area of controversy in the literature regarding the tolerability of mefloquine. Neuropsychiatric disorders include two broad categories of symptoms namely central and peripheral nervous system disorders (including headache, dizziness, vertigo, seizures) and psychiatric disorders (including major psychiatric disorders, affective disorders, anxiety and sleep disturbances). Lobel *et al *[27] found an incidence of strange dreams (25%), insomnia (9%) and dizziness (8.4%) in Peace Corps volunteers using long-term mefloquine prophylaxis similar to those reported by users of chloroquine (corresponding incidence 26%, 6.5%, 10%). No serious neuropsychiatric reactions were causally associated with mefloquine use in this study. Steffen *et al *[57]. reported similar findings in an analysis of tourists (n = 139,164) returning from East Africa. Headache was observed in 6.2% of mefloquine users versus 7.6% of chloroquine/proguanil users, and dizziness, depression and insomnia by 7.6%, 1.8% and 4.2% of mefloquine users versus 5.5%, 1.7% and 6.3% of the chloroquine/proguanil group. In the UK retrospective survey with telephone interviews [58] significantly more neuropsychiatric AE were reported by mefloquine users compared with travellers taking the chloroquine/proguanil combination. Neuropsychiatric events classified as disabling were reported by 0.7% of mefloquine and 0.09% chloroquine/proguanil users, respectively (P = 0.021). Two travellers taking mefloquine (1:607) versus one traveller using chloroquine/proguanil (1:1181) were hospitalized for such events. Controlled studies have shown a significant excess of neuropsychiatric events in mefloquine users versus comparators [22,59].

More recent studies have used databases of electronically recorded prescriptions and diagnoses to define mefloquine exposure and outcome. The analysis of the UK-based General Practice Research Database [60] compared the risk for psychiatric disorders during or after the use of mefloquine with the risk of other anti-malarials; this large study concluded that mefloquine did not increase the first time diagnosis of depression, but may increase the risk of psychosis and anxiety reactions. A second large study in US service members used hospitalization as an objective measure of morbidity and concluded that mefloquine-prescribed personnel were at no increased risk of hospitalization for any disorder including mental disorders and diseases of the nervous system [48].

The precise role of anti-malarial drugs in neuropsychiatric adverse events is difficult to define and the role of travel as a catalyst for such events should be considered together with other confounding factors such as gender predisposition and the use of recreational drugs. The WHO recommends that mefloquine be contraindicated for persons with a personal or family history of psychiatric disorders. In terms of all AE, studies have shown that women are significantly more likely to experience AE [22,54,58,61-63]. This might be due to dose-related toxicity and one study has shown an association between low body weight and a relatively high risk of developing AE during malaria prophylaxis [64]. It might be due to reporting bias, greater compliance with prescription or to gender related differences in drug absorption, metabolism or CNS distribution or gene polymorphisms [53]. Reduced or split dosage in women may provide comparable chemoprophylactic protection and may result in improved tolerability but data are lacking. Because of the long half-life of the drug, mefloquine induced neuropsychiatric adverse events may persist for months, but few data are available on the duration of such events [49]. New innovative research has sought to find biological, neurobiological or pharmacogenetic causes of neuropsychiatric adverse events. This has been made possible by new techniques in neurobiology, neurophysiology and genetic analyses that were hitherto unavailable in the early product life of the drug.

### Mechanisms underlying poor tolerability

Recent research in animal models proposes mechanisms that may explain the neuropsychiatric profile of adverse events associated with mefloquine. Some of the mechanisms that have been proposed to explain neuropsychiatric adverse events with mefloquine include disruption of calcium homeostasis of neuronal cells, inhibition of enzymes such as acetylcholinesterase or butylcholinesterase, inhibition of cellular transport systems (APT-sensitive potassium channel, P-glycoprotein), blockage of receptors (adenosine A2A, p2x7, receptor-mediated spontaneous inhibitory postsynaptic currents) and blockage of intercellular channels (gap junctions) [65]. The phenomenon of "*connexin blockade*" by mefloquine (and other substances) is receiving a lot of attention in the field of neuroscience as a possible mechanism for certain mefloquine associated adverse events. "Connexins" are neuronal gap junction proteins that occur in the brain, lens, retina, and elsewhere. These connexins are assigned numbers according to their molecular weight. The connexins Cx36 and Cx43 are widely distributed in neuronal tissue. With regard to its sensitivity to mefloquine, Cx36 is an important gap junction protein and is thought to be involved in synchronizing rhythmic activity of neurons in several brain regions. Experiments in a rat model show that mefloquine effectively blocks connexin 36 which may explain the biological basis of some of mefloquine's side-effects such as anxiety, confusion, dizziness and other neuropsychiatric effects [66]. A paradoxical effect of connexin 36 blockade is an excitory effect on cerebral seizure-like activity (in rats and mice) as shown recently by Voss *et al *[67].

Most of the studies on possible mechanisms of mefloquine neurotoxicity were conducted at relatively high mefloquine concentrations in animal models such as knockout mice or in rat brain slices and the transfer of knowledge acquired in these studies to healthy humans using mefloquine at chemoprophylaxis dosage still requires the bridging of a large knowledge gap.

The use of genetic analyses to explain a predisposition to mefloquine neuropsychiatric events including seizures is generating warranted interest but careful attention must be paid to confounding factors in order to minimize the chance of spurious associations.

Mefloquine crosses the blood brain barrier in a rat model, and human data shows that there is accumulation in brain tissue particularly of the (+) enantiomer [68]. Mefloquine has two asymmetric carbon atoms and consequently exists as two enantiomers, which are both active against *P. falciparum in vitro*. A study in human volunteers documented that the pharmacokinetic of mefloquine is highly stereospecific. Higher plasma concentration, longer half-live and a higher systemic exposure have been shown for the (-)-enantiomer [69].

Another study investigated the enantioselective kinetics of mefloquine during long term prophylactic treatment [70]. These authors showed a time dependent kinetic behaviour as well as possible enterohepatic recirculation for the (-)-enantiomer and concluded that not only the pharmacokinetic but also other aspects of the disposition and the distribution of both enantiomers are different. An earlier tolerability study aimed to correlate non-serious AE occurring during routine chemoprophylaxis with concentrations of racemic mefloquine, its enantiomers or the carboxylic acid metabolite [62]. The disposition of mefloquine was found to be highly selective but neither the concentrations of enantiomers, nor total mefloquine nor metabolite were found to be significantly related to the occurrence of non-serious AE. In animals a wide and extensive tissue distribution of both enantiomers has been demonstrated, with higher tissue than plasma concentrations. The efflux transporter P-glycoprotein (P-gp) decreases concentration of both enantiomers in tissues such as the brain. In mice the (+)-mefloquine enantiomer seems to be the better substrate for P-gp and it has been therefore more extensively excluded from the blood-brain-barrier than the (-)-enantiomer. The importance of these differences between both enantiomers for the pharmacological activity is currently being researched.

Furthermore, new research shows that genes encoding various receptors, transporters, ion channels and connexins differ between the sexes [71] so that men and women show varying disease profiles and reactions to medications [63] and this may also partially explain the excess of neuropsychiatric adverse events of mefloquine in women. The controlled, double-blind studies comparing mefloquine-associated adverse events with that of the comparator regimens however provide the most objective data [22,54,59,72,73]. Some newer studies using objective, validated psychomotor tests and specialized mood questionnaires such as the "Profile of Mood States" which endeavour to quantify moods and feelings [54] identify an excess of women who experience and report neuropsychiatric type adverse events.

A role has been suggested for the concomitant use of mefloquine and recreational drugs or an interaction between mefloquine and large quantities of alcohol [74], although concomitant use of small quantities of alcohol does not appear to adversely affect tolerability. Children tolerate mefloquine well as do elderly travellers who report significantly fewer AE than younger counterparts [50]. One report suggests that subjects with AE have slower elimination of mefloquine than the population in general. Careful screening of travellers with particular attention to contraindications such as personal or family history of epilepsy/seizures or psychiatric disorders, should minimize the occurrence of serious AE. Up to 10% of the travellers who need malaria chemoprophylaxis may have a contraindication and women are twice as likely as men to have such contra-indications [75]. An analysis of mefloquine prescriptions showed that some 13.8% of prescriptions were issued to individuals who had pre-existing contra-indications [75]. Clearly travel health advisors need to exercise more caution. In those with no contra-indications but where there is a fear of adverse events, some clinicians recommend starting mefloquine three weeks prior to travel to allow for adverse event screening. Some recommend using a split dose (a half tablet twice weekly) for low body weight women. Anecdotal reports suggest positive experience with this approach but no published pharmacokinetic data is available.

### Tolerability of mefloquine according to age profile of users

Mefloquine is considered to be well tolerated in small children where the main adverse events are of gastrointestinal nature, probably associated with the bitter taste of the medication which should be disguised with chocolate or yoghurt.

When mefloquine (Lariam^®^) spontaneously reported adverse events reported to F. Hoffmann-La Roche are categorized by system organ class and age of the mefloquine user, the adverse event profile shows that the neuropsychiatric adverse events dominate across all age groups with the exception of children under two where gastrointestinal events predominate (Table 6). Elderly individuals report lower proportions of neuropsychiatric events compared to the "adolescent" and "adult" categories.

**Table 6:** Adverse event (AE) profile according to age (System Organ Class as a percentage of total reported AE*)

No-SOC Abbrev	INFANT (n = 35) (>1 mth - 2 yrs)	CHILD (n = 212) (>2 yrs - 12 yrs)	ADOLESCENT (n = 233) (>12 yrs - 17 yrs)	ADULT (n = 9896) (>17 yrs - 65 yrs)	ELDERLY (n = 451) (over 65 yrs)
01-INFEC-Total	20.00	8.00	4.30	2.34	4.65

03-BLOOD-Total	5.70	4.70	3.40	1.77	3.76

04-IMMUN-Total	5.70	0.47	0.86	0.45	0.22

05-ENDO-Total	0.00	0.47	0.00	0.16	0.22

06-METAB-Total	0.00	3.00	4.29	2.78	6,2

07-PSYCH-Total	8.50	32.50	40.30	39.48	31.04

08-NERV-Total	17.10	27.30	39.48	35.08	35.90

09-EYE-Total	2.86	5.18	6.86	6.05	4.65

10-EAR-Total	0.00	5.18	6.43	6.55	7.09

11-CARD-Total	5.70	3.70	3.86	7.21	10.64

12-VASC-Total	2.86	1.40	2.14	2.67	4.65

13-RESP-Total	17.10	2.30	5.15	4.04	7.09

14-GASTR-Total	40	25.00	29.18	22.84	29.49

15-HEPAT-Total	0.00	0.94	1.28	1.00	2.66

16-SKIN-Total	14.28	16.98	12.87	12.42	15.52

17-MUSC-Total	0.00	2.83	7.72	5.66	8.65

18-RENAL-Total	0.00	2.83	1.28	1.18	3.99

19-PREG-Total	0.00	0.00	0.86	15.05	0.00

20-REPRO-Total	0.00	0.00	2.15	1.70	0.66

21-CONG-Total	2.86	0.00	0.00	0.92	0.22

22-GENRL-Total	31.42	25.0	18.88	19.64	20.18

23-INV-Total	8.50	6.60	7.30	4.78	8.86

24-INJ&P-Total	14.28	7.50	1.72	1.23	3.54

26-SOCCI-Total	0.00	0.00	0.43	2.13	0.00

02-NEOPL-Total	0.00	0.00	0.00	0.23	0.44

25-SURG -Total	0.00	0.00	0.00	0.18	0.00

Total	100%	100%	100%	100%	100%

* Data on file - F. Hoffmann La Roche, Drug Safety Data Base, MedDRA version 12.1, cut off date August 16^th^, 2010

Key:

01- Infections and infestations

02- Neoplasm benign, malignant and unspecified (incl. cysts and polyps)

03- Blood and lymphatic system disorders

04- Immune system disorders

05- Endocrine disorders

06- Metabolism and nutrition disorders

07- Psychiatric disorders

08- Nervous system disorders

09- Eye disorders

10- Ear and labyrinth disorders

11- Cardiac disorders

12- Vascular disorders

13- Respiratory, thoracic and mediastinal disorders

14- Gastrointestinal disorders

15- Hepatobiliary disorders

16- Skin and subcutaneous tissue disorders

17- Musculoskeletal and connective tissue disorders

18- Renal and urinary disorders

19- Pregnancy, puerperium and perinatal conditions

20- Reproductive system and breast disorders

21- Congenital, familial and genetic disorders

22- General disorders and administration site conditions

23- Investigations

24- Injury, poisoning and procedural complications

25- Surgical and medical procedures

## Conclusion

Mefloquine is an effective anti-malarial (except in clearly defined areas of multi-drug resistance at Thai borders) that has been widely used by a broad spectrum of age groups. Based on the literature reports, studies, national and international guidelines mefloquine can be particularly useful in special populations such as pregnant women, infants and small children, as well as long-term travellers and VFRs, in which efficacy, safety or cost-benefit profile have been well-documented. Careful prescribing of mefloquine with attention to contraindications is essential, as is a clear warning about the potential side effects.

Because many mefloquine-associated adverse events occur early in dosing, during the intake of the initial three tablets, starting mefloquine prophylaxis 2-3 weeks before departure may allow for evaluation of tolerability to the regimen. Stopping the drug with early signs of such events should minimize the severity and duration of adverse events and women may consider the use of the split dose.

Mefloquine has a pivotal position as an important first-line anti-malarial drug and needs to be preserved. For many of its indications, there is currently no replacement available or in the pipeline.

## Conflict of interests

This paper is based on data collated for an F. Hoffmann-La Roche regulatory update. PS was the external consultant who received consultancy fees for preparing the regulatory update. PS has also received research funding and/or speakers' honoraria from GlaxoSmithKlein, F. Hoffmann-La Roche and Pfizer.

MA, LR, MTS, HGR are employees of F. Hoffmann-La Roche, Basel, Switzerland

## Authors' contributions

PS was responsible for the concept and design, acquisition of data, analysis and interpretation of data and the writing of the final manuscript. MA, LR were responsible for certain portions of data acquisition and MA, LR, MTS, HGR critically revised the paper.

All authors have seen and approved this final version.
